# Cold-tolerant bacteria from alpine Rosaceae plants modulate transcriptional responses of apple and strawberry plantlets to freezing stress

**DOI:** 10.3389/fpls.2026.1843975

**Published:** 2026-06-26

**Authors:** Malek Marian, Matteo Buti, Pier Luigi Bianchedi, Ilaria Pertot, Michele Perazzolli

**Affiliations:** 1 Center Agriculture Food Environment (C3A), University of Trento, Trento, Italy; 2 Department of Agriculture, Food, Environmental and Forestry Sciences (DAGRI), University of Florence, Florence, Italy; 3 Research and Innovation Centre, Fondazione Edmund Mach, Trento, Italy

**Keywords:** cold stress, gene expression, RNA-Seq, stress acclimation, stress tolerance, transcriptomic

## Abstract

**Introduction:**

Freezing stress is a frequent challenge in agricultural systems and represents one of the major abiotic constraints on fruit production in Rosaceae crops. Plant-associated microorganisms can support plant survival under cold stress conditions, but little is known about the transcriptional responses stimulated by cold-adapted bacteria in host plants under freezing stress. This study aimed to investigate transcriptional responses activated in apple and strawberry plantlets to freezing stress following the inoculation with two cold-tolerant bacteria isolated from alpine Rosaceae plants.

**Methods:**

Plantlets were treated with sterile 10 mM MgSO_4_ (mock-inoculated) or with a suspension of *Pseudomonas* GRAN103 (apple) and *Duganella* ALCN104 (strawberry) and exposed to non-stress (23 ± 1 °C) or freezing stress (-6 ± 0.5 °C for 3 h) conditions.

**Results:**

Cold-tolerant bacteria isolated from alpine Rosaceae plants decreased electrolyte leakage in apple and strawberry plantlets exposed to freezing stress. Moreover, freezing stress caused distinct transcriptional responses between mock- and bacterium-inoculated plants, as well as between the two crops. In mock-inoculated apple plantlets, freezing stress downregulated transcripts related to amino acid metabolism, lipid metabolism, and secondary metabolism. In contrast, *Pseudomonas* GRAN103- inoculated apple plantlets upregulated genes involved in amino acid and protein metabolism, hormonal signaling, oxidative stress, secondary metabolism, stress response, and transcription regulation. In strawberry, freezing stress downregulated genes involved in transcription, protein and amino acid metabolism in mock-inoculated plantlets. On the other hand, *Duganella* ALCN104-inoculated plantlets upregulated pathways associated with lipid metabolism, secondary metabolism, signal transduction, and transport under freezing stress.

**Discussion:**

The inoculation with *Pseudomonas* GRAN103 and *Duganella* ALCN104 reprograms the transcriptional responses of Rosaceae plants to freezing stress, promoting the upregulation of stress-related pathways that may enhance tolerance to freezing stress.

## Introduction

Freezing stress caused by sub-zero temperatures is a frequent challenge in agricultural systems, and it represents one of the major abiotic constraints to fruit production in Rosaceae crops (Salazar-Gutiérrez et al., 2016). Freezing stress causes devastating damage to the yield and fruit quality of apple and strawberry production across many temperate regions of the European Union, United States, and China (Unterberger et al., 2018; Dalhaus et al., 2020; Chen et al., 2023; Zhang et al., 2025). Climate change is responsible for mild winters and warm springs that promote early spring-related phenological events in plants, increasing the possible occurrence of severe spring frosts and related damage to plants (Ru et al., 2023; Caspersen et al., 2025).

Freezing stress causes substantial damage to vegetative and reproductive tissues of shoots (Salazar-Gutiérrez et al., 2016). At the cellular level, freezing stress causes structural damage to cell membranes, leading to electrolyte leakage and overproduction of reactive oxygen species (ROS), resulting in oxidative damage (Zhang et al., 2025). However, apple and strawberry plants attempt to activate physiological and molecular mechanisms to overcome the impact of freezing stress, such as the accumulation of osmoregulatory substances (e.g., dehydrins, soluble sugars, betaine, and proline), which decrease osmotic pressure and stabilize cell membranes, the activation of antioxidant enzymes (e.g., superoxide dismutase, peroxidase, and catalase), and the accumulation of antioxidants (e.g., ascorbic acid, carotenoids, and flavonoids) to control ROS homeostasis (Li et al., 2023a; Zhang et al., 2025). In particular, apple and strawberry responses to cold stress include the activation of signaling pathways mediated by CRT-binding factor (CBF, also referred to as dehydration responsive element-binding protein 1 or DREB1) (Jia et al., 2026.; Mei et al., 2023; Shen et al., 2023; Yang et al., 2025). The expression of *CBF* genes is rapidly induced upon exposure to cold stress, thereby inducing the expression of cold-responsive (COR) genes and triggering physiological responses (Jia et al., 2026). Moreover, other regulators are involved in cold stress response, such as WRKY and NAM-ATAF1/2-CUC2 (NAC) transcription factors (Mei et al., 2023; Shen et al., 2023; Yang et al., 2025). For example, Shen et al. (2023) demonstrated that MdARF17 plays a positive role in freezing stress tolerance in apple plants by influencing the expression of cold-responsive genes (*MdWRKY33*) and mediating ROS scavenging. Moreover, transcriptomic studies revealed comprehensive information about the expression dynamics of genes and regulatory networks in apple stems (Lee et al., 2023), apple bark (Liang et al., 2020), and strawberry leaves (Zhang et al., 2019) in response to freezing stress.

Plants are associated with highly diverse, complex, and dynamic microbial communities, and plant-associated microorganisms can promote plant growth and survival under cold stress conditions (Su et al., 2015; Allsup et al., 2023; D’Amours et al., 2024; Marian et al., 2022). For example, treatment of strawberry plants with ice nucleation-deficient mutants of *Pseudomonas syringae* and *P. fluorescens* protected strawberry flowers against freezing stress initiated by other ice-nucleation active *P. syringae* (Lindemann and Suslow, 1987). Likewise, cold-adapted bacterial endophytes (*Duganella*, *Erwinia*, *Pseudomonas*, and *Rhizobium* genera) isolated from alpine Rosaceae plants mitigated freezing stress in strawberry seedlings (Marian et al., 2025) and tomato seedlings (*Chryseobacterium* and *Pseudomonas* genera) (Milanese et al., 2025). Moreover, treatments with exogenous phytohormones and chemical inducers improved apple and strawberry freezing and cold stress tolerance, such as abscisic acid, jasmonic acid, brassinosteroids, and ethylene (An et al., 2021, 2022, 2023; Wang et al., 2021; Liu et al., 2025), melatonin and spermidine (Hayat et al., 2022; Ozherelieva et al., 2023; He et al., 2024). Thus, freezing stress frequently causes losses in apple and strawberry yield and quality, but chemical and biological products represent a promising strategy to enhance freezing tolerance in these Rosaceae crops. However, no information is available on transcriptional responses stimulated by cold-adapted bacteria in Rosaceae crops under freezing stress. This study aimed to examine transcriptional regulations activated by freezing stress in apple and strawberry plantlets following the inoculation with cold-tolerant bacteria isolated from alpine Rosaceae plants.

## Materials and methods

### Bacterial isolates and culture conditions

A total of 15 cold-tolerant bacterial endophytes were previously characterized for their ability to mitigate freezing stress in strawberry seedlings (Marian et al., 2025), and they were used in this study (**Supplementary Table 1**). Each isolate was grown overnight (18 h) in liquid R2A medium at 25 °C under orbital shaking at 200 rpm, and bacterial cells were collected by centrifugation (3,500 × g for 10 min) and washing (three times) with sterile 10 mM MgSO_4_ as previously described (Milanese et al., 2025). The bacterial suspension was then adjusted to an optical density at 600 nm (OD_600_) of 0.1, corresponding to 1.0 × 10^8^ CFU mL^-1^ as previously described (Milanese et al., 2025).

### Inoculation of apple plantlets with cold-tolerant bacteria

Apple plantlets (*Malus x domestica*) cultivar Golden Delicious, which is one of the most widely cultivated varieties worldwide, were micropropagated as previously described (Ciccotti et al., 2008). Briefly, an apple shoot (2 cm long) was cut from 6-weeks-old apple plantlets grown *in vitro* and it was transferred into a glass tube for plant tissue culture (30 mm diameter and 150 mm height; Artiglass, Padova, Italy) containing 9 mL of Quoirin Lepoivre 6 medium (macro and micro elements, Duchefa Biochemie, Haarlem, The Netherlands, Quoirin and Lepoivre 1977) supplemented with 30 g L^-1^ sucrose, phytohormones (0.05 mg L^-1^ indole-3-butyric-acid, 1 mg L^-1^ 6-benzylaminopurine, and 0.1 mg L^-1^ gibberellic acid), 1 mL L^-1^ MS vitamins (Duchefa Biochemie), and 7 g L^-1^ agar with pH adjusted to 5.6. Plantlets were grown for two weeks in a growth chamber (16 h/8 h light/dark photoperiod with 23 ± 1 °C during the day and 18 ± 1 °C during the night).

Apple plantlets were treated with 100 µL of sterile 10 mM MgSO_4_ (mock-inoculated) or inoculated with 100 µL of bacterial suspension (1.0 × 10^8^ CFU mL^-1^; bacterium-inoculated) by distributing droplets (5 µL) on each leaf. Apple plantlets were incubated in the growth chamber for four weeks and exposed (freezing-stressed plantlets) or not (non-stressed plantlets) to freezing stress as described by Jiang et al., 2020.

### Inoculation of strawberry plantlets with cold-tolerant bacteria

Seeds of the strawberry (*Fragaria* × *ananassa*) cultivar Fresca (Moles Seeds 261 Ltd, Essex, United Kingdom), which is one of the most widely cultivated varieties worldwide, were germinated as previously described (Ito et al., 2011). Briefly, seeds were stratified at 4 °C for 3 weeks and scarified with cold 96% sulfuric acid for 10 min, followed by five washes (2 min each) with sterile distilled water (SDW). Seeds were surface disinfected with 70% ethanol for 1 min, 1% sodium hypochlorite for 1.5 min, and 70% ethanol for 1 min, followed by five washes with SDW. Surface-disinfected seeds were sown on two layers of filter paper (Whatman, GE Healthcare Life Sciences, Chicago, IL, US) in Petri dishes (90 mm in diameter) moistened with 5 mL of SDW. The plates were incubated in a growth chamber at 23 °C ± 1 °C for four days with a 14 h/10 h light/dark photoperiod. Germinated seeds were transferred to 12-well plates (Greiner-Bio one, Merck, Darmstadt, Germany) containing solid (7 g L^-1^ agar) full-strength Hoagland (2.75 mL well^-1^; pH adjusted to 6.5) (Sigma-Aldrich, Merck), sealed with parafilm, and incubated in the growth chamber for one week. Strawberry plantlets were treated with 100 μL of sterile 10 mM MgSO_4_ (mock-inoculated) or inoculated with a bacterial suspension (bacterium-inoculated) by distributing droplets (5 µL) on the first two leaves (Vogel et al., 2021). Strawberry plantlets were incubated in the growth chamber for four weeks and exposed (freezing-stressed plantlets) or not (non-stressed plantlets) to freezing stress as described by Jiang et al. (2020).

### Exposure of apple and strawberry plantlets to freezing stress and sample collection

Mock-inoculated and bacterium-inoculated apple and strawberry plantlets were exposed (freezing-stressed plantlets) to freezing stress as previously described (Jiang et al., 2020). Briefly, plantlets were placed in a freezing chamber (Versatile Environmental Test Chamber, MLR-351H, Sanyo, Japan) at 4 °C for 30 min in darkness, the temperature was decreased by a cooling rate of 2 °C h^-1^, and plantlets were incubated at –6 °C for 3 h to simulate freezing stress. The temperature of the freezing chamber was increased at a rate of 2 °C h^-1^, and plantlets were incubated at 4 °C for 4 h in darkness. As control, mock-inoculated and bacterium-inoculated plantlets were incubated at 23 °C ± 1 °C in the growth chamber (non-stressed plantlets). Plantlets were then transferred to the growth chamber (23 °C ± 1 °C and 14 h/10 h light/dark photoperiod) for 24 h before sample collection.

### Assessment of electrolyte leakage

Shoot samples of apple and strawberry plantlets exposed to freezing stress were cut and transferred to tubes containing 40 mL of SDW as previously described (Marian et al., 2025). Tubes were incubated overnight (18 h) at 25 °C under orbital shaking at 150 rpm, and the initial electrical conductivity (EC1) was measured using a hand-held conductivity meter (pH CO 1030, pH/Conductivity Tester VWR, Milan, Italy). Samples were autoclaved, and the total conductivity (EC2) was measured. Electrolyte leakage was calculated as follows: (EC1)/(EC2) × 100 (Marian et al., 2025). For apple, five replicates (plantlets) were analyzed for each inoculation condition, and the experiment was carried out twice. For strawberry, six replicates (plantlets) were analyzed for each inoculation condition, and the experiment was carried out three times.

### Statistical analysis

Electrolyte leakage values were normalized by arcsine transformation, followed by pairwise comparison between bacterium-inoculated and mock-inoculated samples with Dunnett’s test (*P* ≤ 0.05) using the DescTools R package (Signorell, 2023). Data were checked for normality and homoscedasticity using Shapiro-Wilk (*P* > 0.05) and Levene’s test (*P* > 0.05) using the rstatix R package (Kassambara, 2023), respectively.

### RNA extraction, library preparation, and sequencing

Shoots of apple and strawberry plantlets treated with 10 mM MgSO_4_ (mock-inoculated) or inoculated (bacterium-inoculated) with the most efficient bacterial isolate (*Pseudomonas* GRAN103 and *Duganella* ALCN104, respectively) were collected in triplicate (pool of three and ten plantlets, respectively) from plantlets exposed (freezing-stressed) or not (non-stressed) to freezing stress. Samples were immediately frozen in liquid nitrogen and stored at -80 °C and RNA extraction was carried out as previously described (Milanese et al., 2025). Frozen shoots were ground to a fine powder in refrigerated sterile stainless jars with liquid nitrogen using a mixer-mill disruptor (MM 400, Retsch, Haan, Germany) at 25 Hz for 10 sec. The total RNA was extracted from each shoot sample (100 mg) using Spectrum Plant Total RNA Kit (Sigma-Aldrich, Merck) with an on-column DNase treatment with the RNase-Free DNase Set (Qiagen, Hilden, Germany). RNA integrity and concentration were assessed with a High Sensitivity RNA ScreenTape kit and TapeStation 4150 instrument (Agilent Technologies, Santa Clara, CA, United States) and a Nanodrop 8000 spectrophotometer (Thermo Fisher Scientific, Waltham, MA, USA), respectively. Library construction and Illumina Sequencing were carried out at Eurofins Genomics (Ebersberg, Germany) following their in-house Inview Transcriptome Discover approach with strand-specific library protocol. This protocol included purification of poly-A containing mRNA molecules, mRNA fragmentation, random primed cDNA synthesis (strand specific), and adapter ligation and adapter-specific PCR amplification. Libraries were sequenced on a Novaseq instrument (Illumina), and paired-end reads of 150 nucleotides were obtained.

### RNA-seq reads mapping and differential expression analyses

RNA-seq reads mapping and differential expression analyses were carried out as previously described (Baldi et al., 2024). Briefly, the quality of RNA-seq raw reads was assessed with FastQC v0.11.9 (Andrews, 2010). Adapter sequences, low-quality bases, and reads shorter than 40 nucleotides were removed using Trimmomatic v0.39 (Bolger et al., 2014) with the following parameters: ILLUMINACLIP:adapters.fa:2:30:10 LEADING:3 TRAILING:3 SLIDINGWINDOW:4:18 MINLEN:40. Apple reads were mapped to *Malus* x *domestica* GDDH13 v1.1 reference assembly (https://www.rosaceae.org/species/malus/malus_x_domestica/genomeGDDH13_v1.1) (Daccord et al., 2017) and strawberry reads were mapped to *Fragaria* x *ananassa* Camarosa Genome Assembly v1.0 (https://www.rosaceae.org/species/fragaria_x_ananassa/genome_v1.0.a1) (Edger et al., 2019) using HiSat2 v2.2.1 (Kim et al., 2019) with default parameters. Read counts were obtained from the alignment files using featureCounts v1.6.0 software (Liao et al., 2014) with default parameters, based on ‘exon’ feature and ‘gene_id’ meta-feature of apple and strawberry annotation files retrieved from the Genome Database for Rosaceae repository (https://www.rosaceae.org/).

Differential expression analysis was carried out using Bioconductor EdgeR v3.40.2 package (Robinson et al., 2009). EdgeR was used to filter out the not active transcripts [a transcript was considered active if reads per million mapping to that transcript (RPM) were >1 in at least two out of the fifteen libraries], to normalize the RNA libraries based on library dimension, and to visualize the multi-dimensional scaling (MDS) plot based on normalized counts. Non-coding RNA genes were excluded, and differential expression analysis was carried out using the likelihood ratio test. Differentially expressed transcripts (DETs) were selected imposing a Log_2_-transformed fold change (LFC) lower than -2 or higher than 2 and a false discovery rate (FDR) lower than 0.05 in four pairwise comparisons for each plant species, such as mock-inoculated freezing-stressed plantlets and mock-inoculated non-stressed plantlets (MkFS *vs.* MkNS) and bacterium-inoculated freezing-stressed plantlets and bacterium-inoculated non-stressed plantlets (BacFS *vs.* BacNS). Venn diagram representations of upregulated and downregulated DETs were visualized using a web tool (http://bioinformatics.psb.ugent.be/webtools/Venn/) to highlight groups of DETs modulated by freezing stress only in mock-inoculated plantlets, bacterium-inoculated plantlets, or in both inoculation conditions.

### Functional annotation and enrichment analysis of differentially expressed transcripts

Homologies against protein NCBI-nr and Swissprot databases and Gene Ontology (GO) annotations of the *Malus x domestica* GDDH13v1.1 and *Fragaria* x *ananassa* Camarosa Genome Assembly v1.0 transcripts were retrieved from the Genome Database for Rosaceae repository (https://www.rosaceae.org/). Cytoscape 3.9.1 (https://cytoscape.org) with the BiNGO 3.0.5 plugin was used for the GO enrichment analyses (Maere et al., 2005). A hypergeometric test with FDR correction at a 0.05 significance level was applied, using the GOSlim_Plants ontology as a simplified version of the GO tailored for plant biology. Enrichment analysis of Kyoto Encyclopedia of Genes and Genomes annotation (KEGG) was carried out using the R package clusterProfiler v4.6.2, and enriched KEGG categories were selected imposing an adjusted p-value lower than 0.05. The top 10 enriched classes were visualized with bubble plots using ggplot2 v3.5.1.

DETs modulated by freezing stress were classified into 14 functional categories (carbohydrate metabolism, energy metabolism, growth and development, hormonal signaling, lipid metabolism, metabolism, oxidative stress, protein and amino acid metabolism, secondary metabolism, signal transduction, stress response, transcription, transport, and uncharacterized) according to the annotation based on the protein descriptions. Heatmaps summarizing expression profiles and putative functions of DETs belonging to functional categories of hormonal signaling, oxidative stress, secondary metabolism, and stress response were obtained with the R package pheatmap (version 1.0.12) (https://cran.r-project.org/web/packages/pheatmap/index.html).

## Results

### Bacterial inoculation affected electrolyte leakage and transcriptional responses of apple and strawberry plantlets under freezing stress

To assess the effect of cold-tolerant bacteria in freezing stress mitigation, electrolyte leakage was assessed in apple and strawberry plantlets inoculated with cold-tolerant bacteria previously isolated from alpine Rosaceae plants (Marian et al., 2025), such as *Duganella* isolates (AFGN201, ALCN104, and GFBS205), *Erwinia* isolates (ARBN102 and GFBS303), *Pseudomonas* isolates (AFAN204, AFDS202, ARAS204, GFAN103, GRAN103, and GRFN102), *Rhizobium* isolates (ALDS107 and ARDN103), and *Sphingomonas* isolates (ARGN302 and GLGS202) (**Table 1**; **Supplementary Figure 1**). Apple and strawberry inoculation with three (*Pseudomonas* GRAN103, *Pseudomonas* ARAS204, and *Pseudomonas* AFDS202) and five (*Duganella* ALCN104, *Duganella* GFBS205, *Erwinia* GFBS303, *Pseudomonas* AFDS202, and *Rhizobium* ALDS107) cold-tolerant bacteria decreases electrolyte leakage compared to mock-inoculated plantlets, respectively. In particular, inoculation with *Pseudomonas* GRAN103 reduced electrolyte leakage to 28.6 ± 4.2% compared with 56.2 ± 14.2% in mock-inoculated apple plantlets. In strawberry plants, inoculation with *Duganella* ALCN104 reduced electrolyte leakage to 15.0 ± 0.8% compared with 32.6 ± 3.9% in mock-inoculated plantlets. Thus, *Pseudomonas* GRAN103 and *Duganella* ALCN104 were the most efficient bacterial strains against freezing stress in apple and strawberry plantlets, respectively, and were selected for transcriptomic analysis (**Supplementary Figure 1**).

**Table 1:** Electrolyte leakage of apple and strawberry plantlets inoculated with cold-tolerant bacterial endophytes and exposed to freezing stress.

Bacterial treatment ^1^	Electrolyte leakage ^2^	
	Apple	Strawberry
Mock-inoculated	56.2 ± 14.2	32.6 ± 3.9
*Duganella* AFGN201	43.0 ± 10.0	23.1 ± 1.4
*Duganella* ALCN104	43.0 ± 10.1	15.0 ± 0.8 *
*Duganella* GFBS205	43.5 ± 12.2	21.7 ± 2.3 *
*Erwinia* ARBN102	54.0 ± 3.9	22.6 ± 3.2
*Erwinia* GFBS303	58.0 ± 5.9	18.2 ± 2.2 *
*Pseudomonas* AFAN204	47.1 ± 12.4	33.0 ± 3.6
*Pseudomonas* AFDS202	38.6 ± 5.4 *	17.5 ± 4.6 *
*Pseudomonas* ARAS204	38.0 ± 4.7 *	24.2 ± 3.0
*Pseudomonas* GFAN103	40.9 ± 9.6	31.4 ± 3.3
*Pseudomonas* GRAN103	28.6 ± 4.2 *	23.6 ± 5.8
*Pseudomonas* GRFN102	44.0 ± 8.7	23.8 ± 1.3
*Rhizobium* ALDS107	72.1 ± 11.8	21.7 ± 2.4 *
*Rhizobium* ARDN301	47.2 ± 7.3	22.8 ± 0.5
*Sphingomonas* ARGN302	59.3 ± 11.2	24.5 ± 2.0
*Sphingomonas* GLGS202	50.3 ± 11.3	25.5 ± 1.4

^1^ Apple and strawberry plantlets were treated with MgSO_4_ (mock-inoculated) or inoculated with cold-tolerant bacteria isolated from alpine plants (**Supplementary Table 1**) and exposed to freezing stress.

^2^ Electrolyte leakage was assessed in apple and strawberry shoots collected 24 h after exposure to freezing stress. Five and six replicates (plantlets) were analyzed for each inoculation condition of apple and strawberry plantlets, respectively. The experiment was carried out twice and three times for apple and strawberry plantlets, respectively. For each plant species, asterisks indicate significant differences in the pairwise comparisons between bacterium-inoculated and mock-inoculated samples according to the Dunnett’s test (*P* ≤ 0.05).

RNA-Seq analysis was carried out on shoot samples of mock- and bacterium-inoculated plants, and a total of 631,238,877 and 651,983,506 paired-end raw reads were obtained for apple and strawberry samples, respectively, and more than 96% of them were retrieved after quality filtering (**Supplementary Table 2**). From 95.04% to 95.55% of filtered paired-end reads aligned to the apple genome, and from 95.12% to 95.82% aligned to the strawberry genome (**Supplementary Table 2**). MDS plots showed appropriate clustering of replicates, and separation between freezing-stressed and non-stressed samples in the first dimension of apple data and in the second dimension of strawberry data (**Supplementary Figure 2**), according to expression levels of apple (**Supplementary Table 3**) and strawberry transcripts (**Supplementary Table 4**).

**Figure 1:**
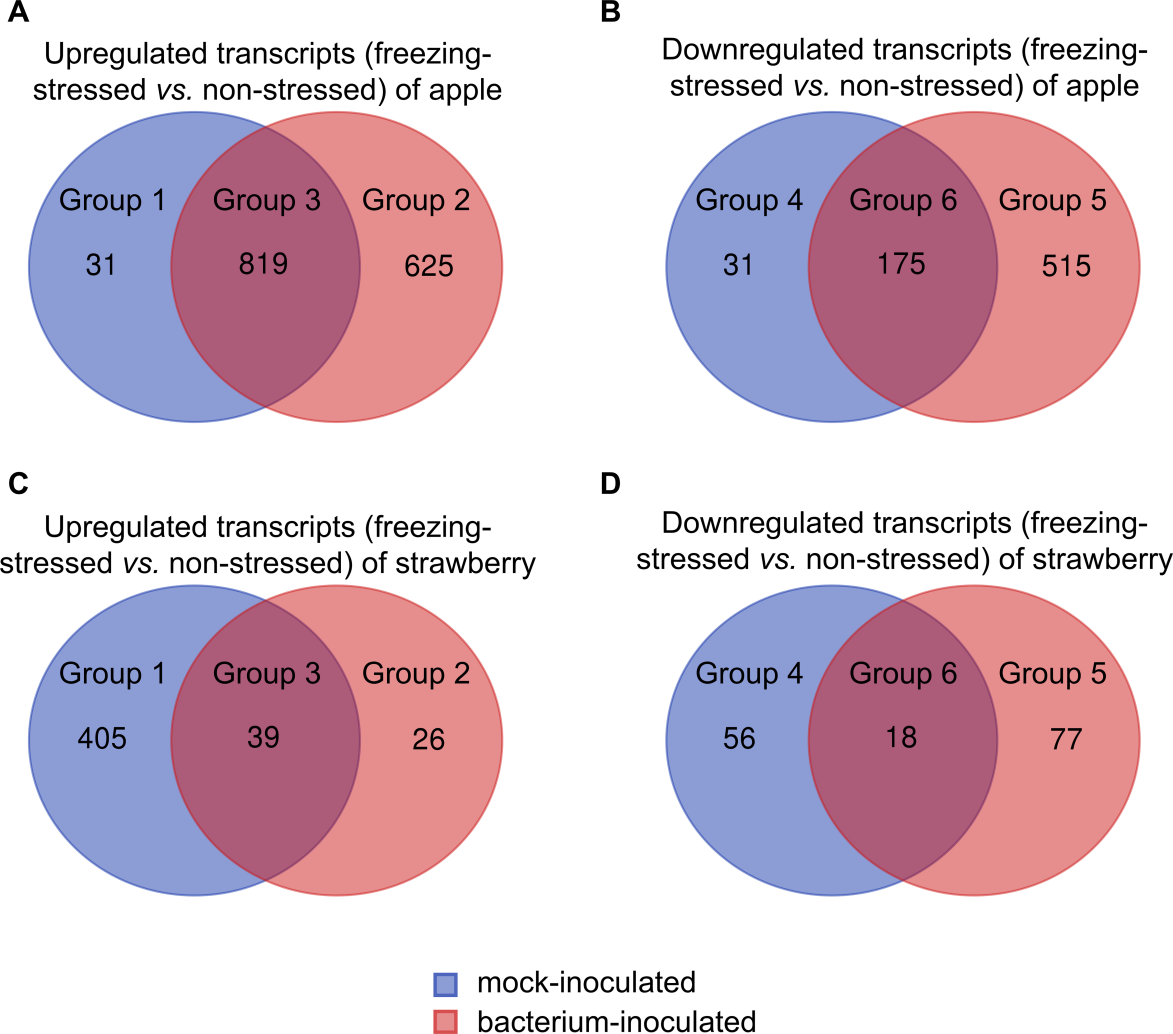
Distribution of transcripts with significant changes in abundance. Venn diagrams summarize differentially expressed transcripts (DEGs) of apple **(A, B)** and strawberry **(C, D)** plantlets treated with 10 mM MgSO_4_ (mock-inoculated) or inoculated (bacterium-inoculated) with *Pseudomonas* GRAN103 and *Duganella* ALCN104, respectively, and exposed (freezing-stressed) or not (non-stressed) to freezing stress. DEGs were grouped into transcripts upregulated or downregulated by freezing stress in mock-inoculated plantlets (group 1 and group 4, respectively), in bacterium-inoculated plantlets (group 2 and group 5, respectively), and in both inoculation conditions (group 3 and group 6, respectively).

Differential expression analysis of apple plantlets highlighted 1,056 DETs in mock-inoculated samples and 2,134 DETs in *Pseudomonas* GRAN103-inoculated samples, imposing an FDR lower than 0.05 and an LFC higher than 2 or lower than -2 in the pairwise comparison between freezing-stressed and non-stressed samples (**Supplementary Tables 5**, **S6**). In particular, upregulated and downregulated apple DETs were classified into those modulated by freezing stress exclusively in mock-inoculated plantlets (groups 1 and 4, respectively), exclusively in *Pseudomonas* GRAN103-inoculated plantlets (groups 2 and 5, respectively), or in both inoculation conditions (groups 3 and 6, respectively; **Figures 1A, B**). In strawberry plantlets, differential expression analysis revealed 518 DETs in mock-inoculated samples and 60 DETs in *Duganella* ALCN104-inoculated samples, imposing an FDR lower than 0.05 and an LFC higher than 2 or lower than -2 in the pairwise comparison between freezing-stressed and non-stressed samples (**Supplementary Tables 5**, **S7**). The lower number of DETs in strawberry compared to apple samples could be due to the greater dispersion observed in the MDS plots (**Supplementary Figure 2**). Upregulated and downregulated strawberry DETs were classified in those modulated by freezing stress in mock-inoculated plantlets (groups 1 and 4, respectively), *Duganella* ALCN104-inoculated plantlets (groups 2 and 5, respectively), or both inoculation conditions (groups 3 and 6, respectively; **Figures 1C, D**). A large fraction of apple DETs was modulated (625 upregulated and 515 downregulated transcripts) in *Pseudomonas* GRAN103-inoculated plantlets, as a possible active response of bacterium-inoculated plants to freezing stress. Conversely, strawberry DETs were mainly modulated (405 upregulated and 56 downregulated transcripts) in mock-inoculated plantlets, indicating a minor effect of *Duganella* ALCN104 inoculation or a more targeted response.

### Transcriptional responses of bacterium-inoculated apple plantlets to freezing stress included the activation of processes associated with stress mitigation

**Figure 2:**
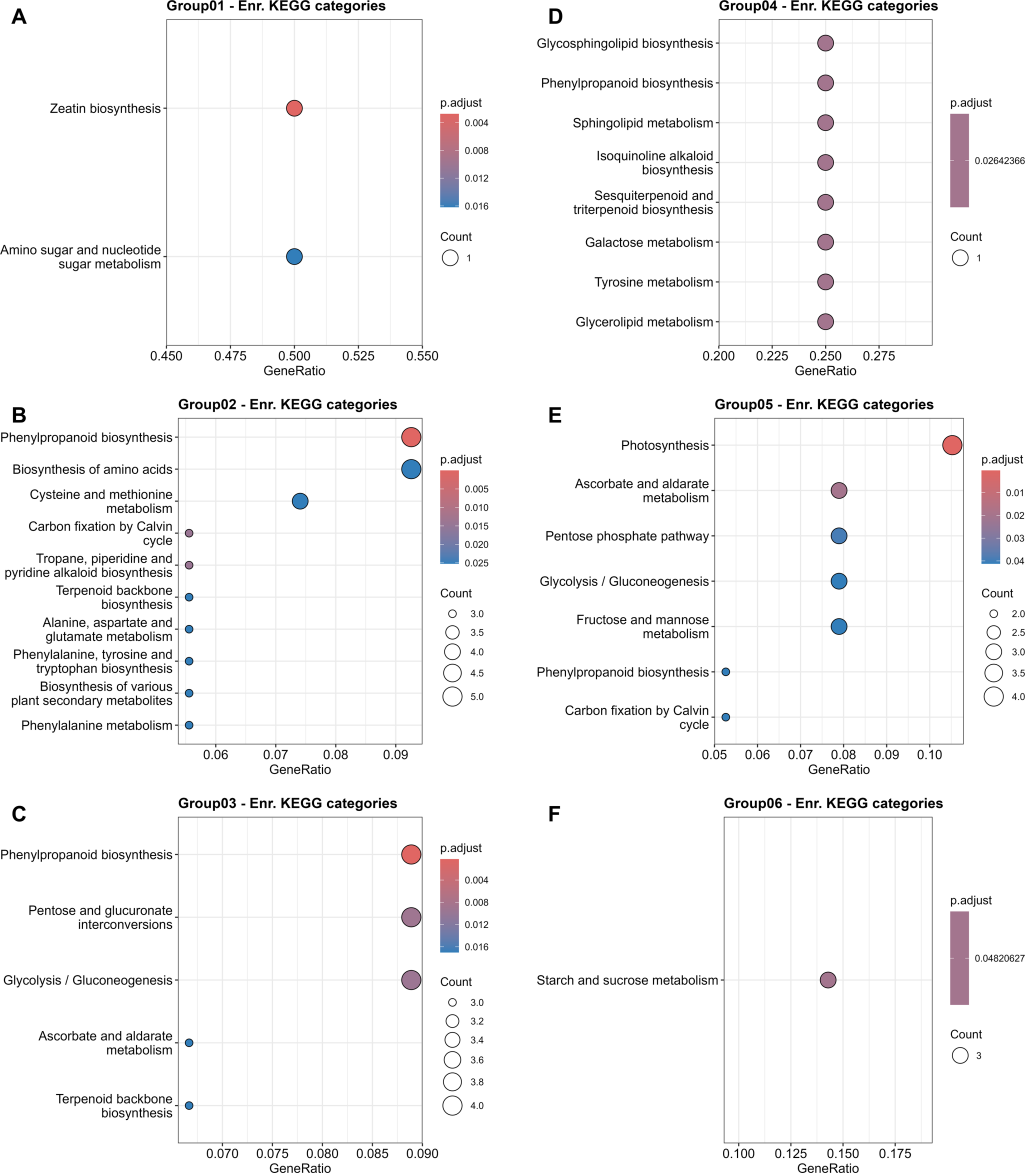
KEGG enrichment analysis of apple differentially expressed transcripts (DETs). Bubble diagrams displaying the top enriched KEGG categories of apple transcripts upregulated [group 1; **(A)**] or downregulated [group 4; **(D)**] by freezing stress only in mock-inoculated plantlets, transcripts upregulated [group 2; **(B)**] or downregulated [group 5; **(E)**] by freezing stress only in *Pseudomonas* GRAN103-inoculated plantlets, and transcripts upregulated [group 3; **(C)**] or downregulated [group 6; **(F)**] by freezing stress in both inoculation conditions. Enriched KEGG categories are shown for mock-inoculated (top), bacterium-inoculated (middle), and both (bottom). The Y axis represents the enriched KEGG categories, while the X axis represents the transcript ratio of DETs compared to apple transcripts belonging to the same KEGG category. The dot size represents the number of DETs for each enriched KEGG category, and dot color represents the adjusted p-value for each enriched KEGG category according to the color scale legend.

KEGG enrichment analysis of apple DETs revealed the enrichment of zeatin biosynthesis and carbohydrate metabolism in transcripts upregulated by freezing stress exclusively in mock-inoculated plantlets (group 1; **Figure 2A**), and processes related to amino acid metabolism and secondary metabolism (e.g., alkaloids, phenylpropanoids, and terpenoids) in transcripts upregulated by freezing stress exclusively in *Pseudomonas* GRAN103-inoculated plantlets (group 2; **Figure 2B**). Apple DETs upregulated by freezing stress in both inoculation conditions were enriched in processes related to carbohydrate metabolism (e.g., glycolysis, gluconeogenesis, and pentose metabolism) and secondary metabolism (e.g., phenylpropanoids and terpenoids) (group 3; **Figure 2C**). Apple DETs downregulated by freezing stress exclusively in mock-inoculated plantlets were enriched in processes related to amino acid metabolism (e.g., tyrosine), lipid metabolism, secondary metabolism (e.g., alkaloids, phenylpropanoids, and terpenoids) (group 4; **Figure 2D**), while those exclusively downregulated in *Pseudomonas* GRAN103-inoculated plantlets were enriched in processes related to carbohydrate metabolism (e.g., fructose metabolism, glycolysis, gluconeogenesis, and pentose metabolism) and secondary metabolism (e.g., phenylpropanoids) (group 5; **Figure 2E**). Moreover, starch and sucrose metabolism were enriched in DETs downregulated by freezing stress in both inoculation conditions (group 6; **Figure 2F**).

**Figure 3:**
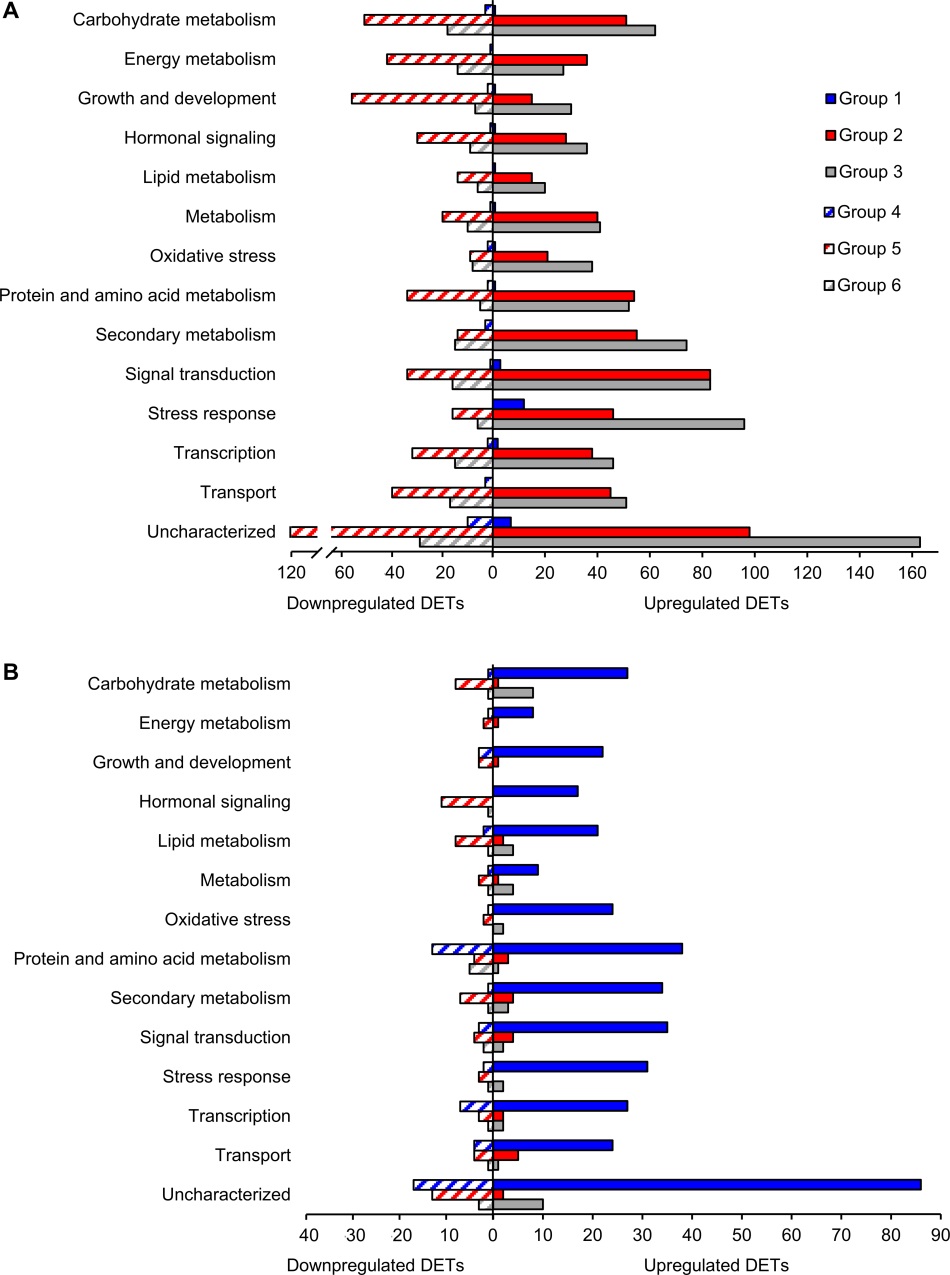
Functional annotation of differentially expressed transcripts (DETs) of apple **(A)** and strawberry **(B)** plantlets. Upregulated (solid bars) and downregulated (stripped bars) DETs (Log2-transformed fold change lower than -2 or higher than 2, and false discovery rate lower than 0.05) of apple **(A)** and strawberry **(B)** plantlets treated with 10 mM MgSO_4_ (mock-inoculated; Mk) or inoculated (bacterium-inoculated; Bac) with *Pseudomonas* GRAN103 and *Duganella* ALCN104, respectively, and exposed (freezing-stressed; FS) or not (non-stressed; NS) to freezing stress were grouped in transcripts upregulated (group 1) or downregulated (group 4) by freezing stress only in mock-inoculated plantlets, transcripts upregulated (group 2) or downregulated (group 5) by freezing stress only in bacterium-inoculated plantlets, and transcripts upregulated (group 3) or downregulated (group 6) by freezing stress in both inoculation conditions. Numbers of upregulated and downregulated DETs are reported for each functional category, according to the annotation based on the protein description.

**Figure 4:**
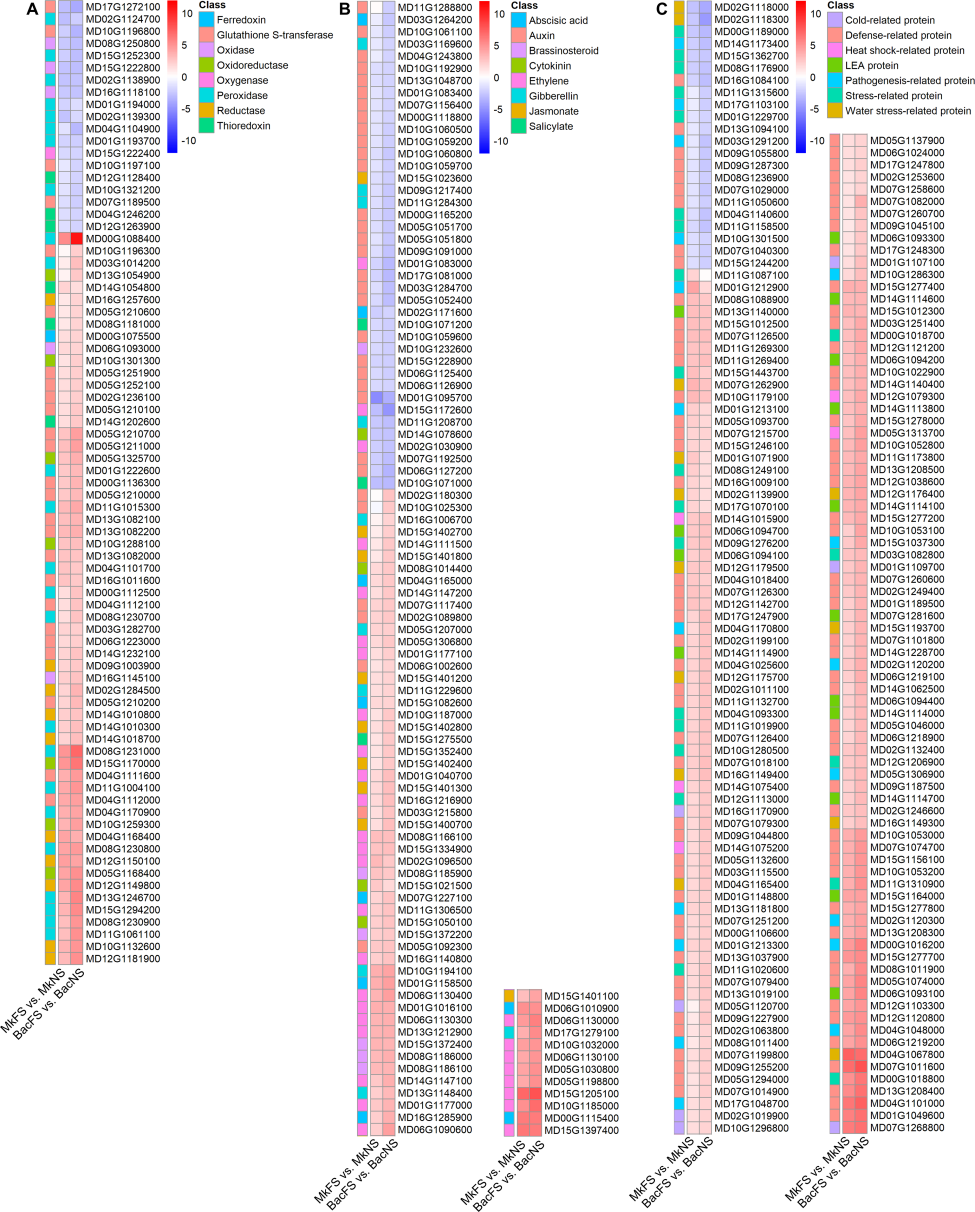
Heatmaps of differentially expressed transcripts (DETs) modulated by freezing stress in apple plantlets and belonging to functional categories of oxidative stress **(A)**, hormonal signaling **(B)**, and stress response **(C)**. Log_2_-transformed fold change values (color legend; LFC) of apple DETs are reported for each pairwise comparison of mock-inoculated freezing-stressed plantlets and mock-inoculated non-stressed plantlets (MkFS *vs.* MkNS), and bacterium-inoculated freezing-stressed plantlets and bacterium-inoculated non-stressed plantlets (BacFS *vs.* BacNS). Putative functions (color legend) of DETs were assigned according to the protein descriptions.

Functional annotation of apple DETs showed that transcripts upregulated (group 1) and downregulated (group 4) by freezing stress exclusively in mock-inoculated plantlets were mainly associated with the functional categories of stress response (**Figure 3A**; **Supplementary Table 6**). Moreover, apple transcripts upregulated (group 2) and downregulated (group 5) by freezing stress exclusively in *Pseudomonas* GRAN103-inoculated plantlets were mainly associated with the functional categories of carbohydrate metabolism, protein and amino acid metabolism, secondary metabolism, and signal transduction; while those modulated in both inoculation conditions (groups 3 and 6) were mainly associated with carbohydrate metabolism, secondary metabolism, signal transduction, and stress response, although a large fraction of DETs were associated with uncharacterized functions. In particular, DETs related to oxidative stress response were modulated by freezing stress in mock-inoculated apple plantlets (one transcript in group 1 and two transcripts in group 4) and *Pseudomonas* GRAN103-inoculated plantlets (21 transcripts in group 2 and nine transcripts in group 5), or in both inoculation conditions (38 transcripts in group 3 and eight transcripts in group 6), such as one ferredoxin, 25 glutathione S-transferase, five oxidase, seven oxidoreductase, one oxygenase, 24 peroxidase, ten reductase, and six thioredoxin genes (**Figure 4A**; **Supplementary Table 6**). Freezing stress modulated the expression of transcripts related to hormonal signaling in mock-inoculated plantlets (one transcript in group 1 and one transcript in group 4), *Pseudomonas* GRAN103-inoculated plantlets (28 transcripts in group 2 and 30 transcripts in group 5), or in both inoculation conditions (39 transcripts in group 3 and nine transcripts in group 6), such as nine abscisic acid-, 33 auxin-, six brassinosteroid-, four cytokinin-, 31 ethylene-, ten gibberellin-, nine jasmonate-, and three salicylate-related genes (**Figure 4B**; **Supplementary Table 6**). Moreover, the expression of transcripts related to stress response was modulated by freezing stress in mock-inoculated plantlets (12 transcripts in group 1 and no transcripts in group 4), *Pseudomonas* GRAN103-inoculated plantlets (46 transcripts in group 2 and 16 transcripts in group 5), or in both inoculation conditions (96 transcripts in group 3 and six transcripts in group 6), such as transcripts encoding cold- (seven transcripts), defense- (95 transcripts), heat shock- (six transcripts), late embryogenesis abundant- (LEA; 15 transcripts), pathogenesis- (18 transcripts), stress- (22 transcripts), and water stress- (13 transcripts) related proteins (**Figure 4C**; **Supplementary Table 6**).

### Transcriptional responses of bacterium-inoculated strawberry plantlets to freezing stress included the activation of processes associated with stress mitigation

**Figure 5:**
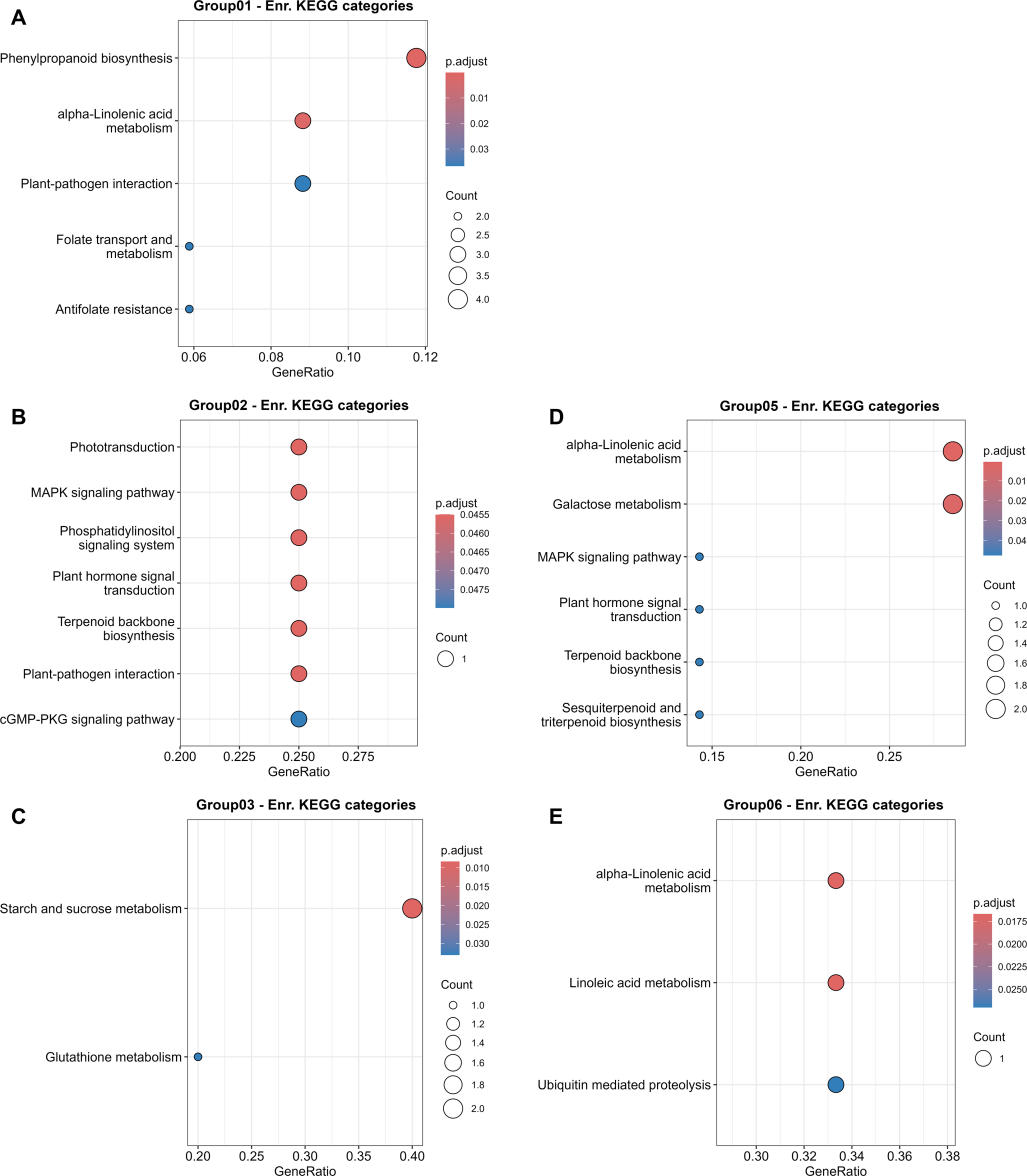
KEGG enrichment analysis of strawberry differentially expressed transcripts (DETs). Bubble diagrams displaying the top enriched KEGG categories grouped in transcripts upregulated [group 1; **(A)**] by freezing stress only in mock-inoculated plantlets, transcripts upregulated [group 2; **(B)**] or downregulated [group 5; **(D)**] by freezing stress only in *Duganella* ALCN104-inoculated plantlets, and transcripts upregulated [group 3; **(C)**] or downregulated [group 6; **(E)**] by freezing stress in both inoculation conditions. The Y axis represents the enriched KEGG categories, while the X axis represents the transcript ratio of DETs compared to strawberry transcripts belonging to the same KEGG category. The dot size represents the number of DETs for each enriched KEGG category, and dot color represents the adjusted p-value for each enriched KEGG category according to the color scale legend. No enriched KEGG categories were found for transcripts downregulated by freezing stress only in mock-inoculated plantlets (group 4).

In strawberry, KEGG enrichment analysis revealed the enrichment of defense-related processes, secondary metabolism (e.g., folate and phenylpropanoids), and lipid metabolism (e.g., linoleic acid) in transcripts upregulated by freezing stress exclusively in mock-inoculated plantlets (group 1; **Figure 5A**). Categories of defense-related processes, secondary metabolism (e.g., terpenoids), and signal transduction (e.g., plant hormone, phosphatidyl inositol, and MAPK signaling pathways) were enriched in transcripts upregulated by freezing stress exclusively in *Duganella* ALCN104-inoculated plantlets (group 2; **Figure 5B**), while processes related to glutathione, starch, and sucrose metabolism were enriched in transcripts upregulated by freezing stress in both inoculation conditions (group 3; **Figure 5C**). Strawberry DETs downregulated by freezing stress exclusively in *Duganella* ALCN104-inoculated plantlets were enriched in processes related to carbohydrate metabolism (e.g., galactose), lipid metabolism (e.g., linoleic acid), secondary metabolism (e.g., terpenoids), and signal transduction (e.g., plant hormone and MAPK signaling pathways; group 5; **Figure 5D**), while DETs downregulated by freezing stress in both inoculation conditions were enriched in processes related to lipid metabolism and protein metabolism (group 6; **Figure 5E**).

**Figure 6:**
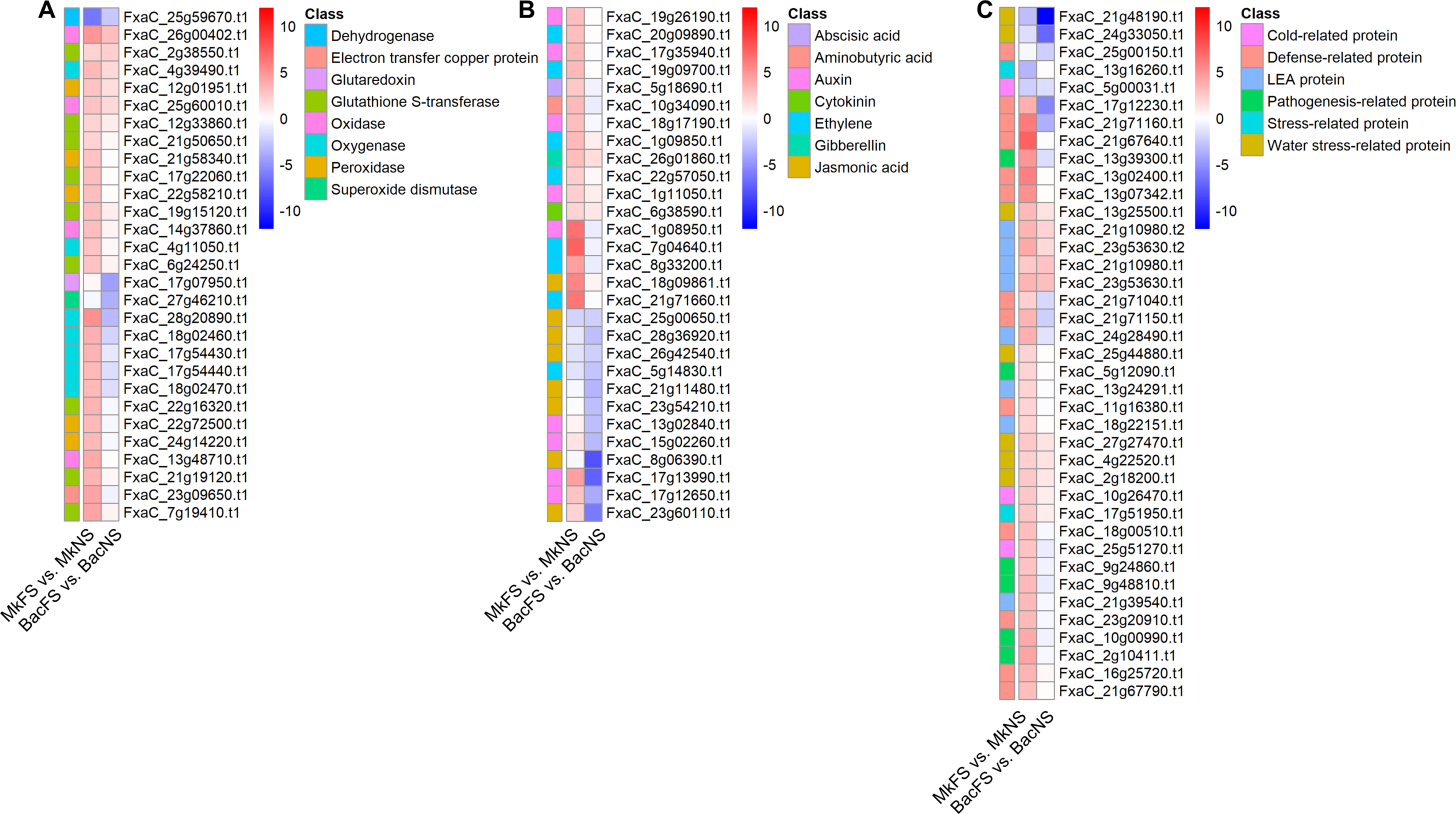
Heatmaps of differentially expressed transcripts (DETs) modulated by freezing stress in strawberry plantlets and belonging to functional categories of oxidative stress **(A)**, hormonal signaling **(B)**, and stress response **(C)**. Log_2_-transformed fold change values (color legend; LFC) of strawberry DETs are reported for each pairwise comparison of mock-inoculated freezing-stressed plantlets and mock-inoculated non-stressed plantlets (MkFS *vs.* MkNS), and bacterium-inoculated freezing-stressed plantlets and bacterium-inoculated non-stressed plantlets (BacFS *vs.* BacNS). Putative functions (color legend) of DETs were assigned according to the protein descriptions.

Although strawberry DETs with unknown functions were found by gene annotation, transcripts upregulated (group 1) and downregulated (group 4) by freezing stress exclusively in mock-inoculated plantlets were mainly associated with the functional categories of protein and amino acid metabolism, and transcription (**Figure 3B**; **Supplementary Table 7**). Strawberry transcripts modulated (groups 2 and 5) by freezing stress exclusively in *Duganella* ALCN104-inoculated plantlets were mainly associated with lipid metabolism, secondary metabolism, signal transduction, and transport; while those modulated in both inoculation conditions (groups 3 and 6) were mainly associated with carbohydrate metabolism, protein and amino acid metabolism. In strawberry plantlets, DETs related to oxidative stress response were upregulated by freezing stress in mock-inoculated samples (23 transcripts in group 1) or in both inoculation conditions (two transcripts in group 3), such as nine glutathione S-transferase, four oxidase, seven oxygenase, and five peroxidase genes (**Figure 6A**; **Supplementary Table 7**). Moreover, freezing stress modulated the expression of transcripts related to hormonal signaling in mock-inoculated plantlets (15 transcripts in group 1) and *Duganella* ALCN104 plantlets (five transcripts in group 5), such as one abscisic acid-, nine auxin-, one cytokinin-, eight ethylene-, one gibberellin-related genes (**Figure 6B**, **Supplementary Table 7**), as well as transcripts related to stress response in mock-inoculated plantlets (32 transcript in group 1 and two transcripts in group 4), *Duganella* ALCN104-inoculated plantlets (three transcripts in group 5), or in both inoculation conditions (two transcripts in group 3 and one transcript in group 6), such as genes encoding cold- (three transcripts), defense- (14 transcripts), LEA- (eight transcripts), pathogenesis- (six transcripts), stress- (two transcripts), and water stress- (seven transcripts) related proteins (**Figure 6C**; **Supplementary Table 7**).

## Discussion

In this study, we showed that bacterial inoculation of apple plantlets (*Pseudomonas* GRAN103, *Pseudomonas* ARAS204, and *Pseudomonas* AFDS202) and strawberry plantlets (*Duganella* ALCN104, *Duganella* GFBS205, *Erwinia* GFBS303, *Pseudomonas* AFDS202, and *Rhizobium* ALDS107) with cold-tolerant bacteria decreased electrolyte leakage under freezing stress, suggesting enhanced freezing stress tolerance compared to mock-inoculated plantlets. A decrease in electrolyte leakage has previously been reported under freezing and cold stress conditions in apple or strawberry plants treated with exogenous phytohormones and chemical inducers, such as abscisic acid, jasmonic acid, brassinosteroids, and ethylene (An et al., 2021, 2022, 2023; Wang et al., 2021; Liu et al., 2025), as well as melatonin and adaptogenic preparations (natural plant complex), and spermidine (Hayat et al., 2022; Ozherelieva et al., 2023; He et al., 2024), indicating consistent effects of bacterial and chemical treatments in cold stress mitigation.

Transcriptional responses to freezing stress were affected by inoculation with *Pseudomonas* GRAN103 and *Duganella* ALCN104, suggesting that cold-tolerant bacteria can promote the upregulation of stress-related pathways, enhancing tolerance to freezing stress of apple and strawberry plants. In apple, mock-inoculated plantlets responded to freezing stress mainly with the downregulation of genes related to amino acid metabolism (e.g., tyrosine metabolism), lipid metabolism, and secondary metabolism (e.g., alkaloids, phenylpropanoids, and terpenoids), reflecting the negative impacts of freezing stress. On the other hand, freezing-stress response in *Pseudomonas* GRAN103-inoculated plantlets involved the upregulation of transcripts related to the metabolism of amino acids and secondary metabolites (e.g., alkaloids, phenylpropanoids, and terpenoids), suggesting the activation of tolerance strategies to freezing stress in bacterium-inoculated plants. In particular, genes associated with protein and amino acid metabolism and γ-aminobutyric acid pathway, were upregulated by freezing stress exclusively in *Pseudomonas* GRAN103-inoculated plantlets, such as glutamate dehydrogenase, glutamate decarboxylase, glutamate receptor, aspartate aminotransferase, aspartate-semialdehyde dehydrogenase alanine aminotransferase, serine carboxypeptidase, inositol polyphosphate 5-phosphatase, adenosylhomocysteinase, homocysteine S-methyltransferase, S-adenosylmethionine-dependent methyltransferase, and S-adenosylmethionine synthases. Moreover, genes involved in protein folding and protein turnover were upregulated by freezing stress exclusively in *Pseudomonas* GRAN103-inoculated plantlets, such as cysteine oxidase, luminal-binding protein, ubiquitin-protein ligase, and tryptophan aminotransferase, suggesting the potential involvement of amino acid metabolism in the freezing tolerance stimulated by *Pseudomonas* GRAN103 inoculation. Changes in amino acid content (e.g., alanine, glycine, proline, and serine) are known to enhance freezing and cold tolerance in plants (Dhont et al., 2006; Xu et al., 2020), and the above mentioned genes associated with amino acid metabolism were suggested as one of key markers for freezing and cold tolerance in various plants including apple (Zhao et al., 2021; Wang et al., 2022; Yu et al., 2025; Sun et al., 2025; Li et al., 2018). Moreover, genes associated with secondary metabolism were upregulated by freezing stress in *Pseudomonas* GRAN103-inoculated apple plantlets, including genes involved in the production of coumarin and indole-derived compounds, suggesting the potential role of secondary metabolites in freezing stress tolerance stimulated by *Pseudomonas* GRAN103 inoculation. Transcriptomic studies have previously associated transcriptional regulations of secondary metabolism with freezing and cold stress tolerance of plants, including apples and other Rosaceae plants (Liang et al., 2020; Lee et al., 2023; Kong et al., 2024; Yu et al., 2025). In particular, phenylpropanoid biosynthesis is responsible for the production of flavonoids, lignins, and other phenolic compounds that play important roles in plant freezing tolerance (Li et al., 2025). In agreement with our findings, the activation of phenylpropanoid biosynthetic pathways (e.g., lignin, flavonoids, and coumarins) has been identified as a key mechanism for apple and peach tolerance to freezing stress (Li et al., 2023a; Yu et al., 2023).

Plant hormones play essential roles in the regulation of cold stress response (An et al., 2021, 2022, 2023; Wang et al., 2021; Liu et al., 2025), and some genes related to hormonal signaling (e.g., abscisic acid, auxins, ethylene, and jasmonic acid) were upregulated by freezing stress exclusively in *Pseudomonas* GRAN103-inoculated apple plantlets. Among them, genes with regulatory roles in hormonal pathways were found, such as abscisic acid receptor and abscisic acid hydroxylase. In addition, we found genes encoding auxin-binding and auxin-responsive proteins, WAT related protein, and indole-3-acetic acid-amido synthetase, suggesting the stimulation of auxin pathways in *Pseudomonas* GRAN103-inoculated plantlets to modulate freezing stress response. It was previously reported that the expression of an auxin-amido synthetase-encoding gene (*MdGH3*) positively regulates cold tolerance and promotes anthocyanin accumulation in apple plants (Jia et al., 2026). Genes encoding ethylene-responsive transcription factors (ERFs), 1-aminocyclopropane-1-carboxylate synthase, and 1-aminocyclopropane-1-carboxylate oxidase were upregulated by freezing stress mainly in *Pseudomonas* GRAN103-inoculated plantlets, and ethylene is known to play a positive role in freezing stress tolerance in apple (Jia et al., 2026), supporting the role of *Pseudomonas* GRAN103 in stimulating hormone-mediated freezing stress response.

Genes related to functional categories of oxidative stress and stress response were mainly upregulated in *Pseudomonas* GRAN103-inoculated plantlets, such as genes encoding glutathione S-transferases, oxidoreductases, peroxidases, reductases, cold-related proteins, heat shock-related proteins, late embryogenesis abundant (LEA) proteins, stress-related proteins, and water stress-related protein. Responses to cold stress are known to be associated with transcriptional regulations of ROS scavenging enzymes and stress-related proteins (e.g., heat shock proteins, LEA proteins, and dehydrins) (Gusain et al., 2023; Wang et al., 2024; Roychowdhury et al., 2025), suggesting that *Pseudomonas* GRAN103 inoculation can stimulate the expression of cold-stress-related pathways in apple plantlets. Moreover, genes associated with transcription regulation upregulated in *Pseudomonas* GRAN103-inoculated plantlets include WRKY, MYB, NAC domain, and C2H2L domain, and zinc finger proteins. Studies on apple plants reported WRKY, NAC, and zinc finger proteins as key positive regulators in freezing and cold stress tolerance by controlling the CBF pathway (Li et al., 2023b; Mei et al., 2023; Shen et al., 2023; Liu et al., 2024; Yang et al., 2025). Likewise, MYB4 and MYB108 have been implicated in freezing and cold stress tolerance in apple and other Rosaceae plant species by regulating phenylpropanoid metabolism and hormone signaling (Dong et al., 2021; Hao et al., 2021; Zhou et al., 2021; Yao et al., 2022). In addition to the specific response of mock- and *Pseudomonas* GRAN103-inoculated plantlets to freezing stress, a large fraction of apple genes were upregulated or downregulated in both inoculation conditions, suggesting that consistent transcriptional responses to freezing stress partially occurred apple plantlets, involving carbohydrate metabolism (e.g., glycolysis, gluconeogenesis, pentose metabolism, starch and sucrose metabolism), secondary metabolism (e.g., phenylpropanoids and terpenoids), and signal transduction.

In strawberry, mock-inoculated plantlets responded to freezing stress mainly with the downregulation of genes related to transcription, protein and amino acid metabolism, corroborating the negative impacts of freezing stress on Rosaceae plants in our study. On the other hand, *Duganella* ALCN104-inoculated strawberry plantlets upregulated genes related to lipid metabolism, secondary metabolism (e.g., terpenoids), signal transduction (e.g., plant hormone, phosphatidyl inositol, and MAPK signaling pathways), and transport. In particular, genes related to fatty acid metabolism (e.g., desaturase and dehydrodolichyl diphosphate synthases), secondary metabolism (e.g., germacrene D synthase, anthocyanidin reductase, and anthocyanidin 3-O-glucosyltransferase), and signal transduction were upregulated by freezing stress in *Duganella* ALCN104-inoculated plantlets. Likewise, previous studies showed that genes encoding fatty acid desaturase, germacrene D synthase, and anthocyanidin 3-O-glucosyltransferase were involved in the freezing and cold tolerance of various plants, including strawberry and apple, through the biosynthesis of unsaturated lipids and phenylpropanoids (Hashempour et al., 2019; Zhang et al., 2019; Zhao et al., 2021; Han et al., 2025; Yousefi et al., 2026). Genes encoding calcium-binding proteins (i.e., CML19, CML41, CML47) and G-type lectin S-receptor-like serine/threonine-protein kinase were upregulated by freezing stress in *Duganella* ALCN104 inoculated plantlets, suggesting their importance in freezing stress tolerance of strawberry. Calcium acts as a key signaling mediator, regulating stress perception and downstream responses to enhance cold tolerance in strawberry and apple plants (Feng et al., 2013; Dong et al., 2020). Likewise, receptor-like protein kinases have been shown to be critical in perceiving external signals and were reported as one of the potential mechanisms involved in the cold stress tolerance of grapevine flowers (Sawicki et al., 2019). However, some strawberry genes were upregulated or downregulated in both inoculation conditions, including those involved in protein, amino acid, glutathione, starch, and sucrose metabolism, and these responses partially overlapped with transcriptional responses in mock- and bacterium-inoculated plants under freezing stress. Moreover, KEGG enrichment results in our study associated with pathways represented by a single DETs should be interpreted with caution, as statistical enrichment in pathways with limited annotated genes may provide restricted biological support. Differences between the two crops cannot be attributed cleanly to species biology alone, because apple and strawberry differ in propagation system, growth medium, photoperiod, and inoculated bacterial isolates. Strong correlation between RNA-Seq and qPCR data was previously well demonstrated (SEQC/MAQC-III Consortium, 2014), and recent RNA-Seq papers are not reporting qPCR validations (Garg et al., 2023; Negussu et al., 2023; Vergata et al., 2023; Basso et al., 2024; Buti et al., 2025; Chen et al., 2026). Future research should therefore be conducted to validate the modulation of apple and strawberry genes under freezing stress using qPCR analysis across independent biological replicates and experimental conditions.

## Conclusions

This study demonstrated that inoculation with *Pseudomonas* GRAN103 and *Duganella* ALCN104 can mitigate freezing stress in apple and strawberry plantlets, respectively, decreasing electrolyte leakage and reprogramming the transcriptional responses to freezing stress. In particular, bacterium-inoculated apple and strawberry plantlets upregulated genes involved in cold stress-related pathways to a greater extent than mock-inoculated plantlets, including amino acid and protein metabolism, lipid metabolism, hormonal signaling, oxidative stress, secondary metabolism, signal transduction, stress response, and transcription regulation. Future research should be carried out to better understand the involvement of apple and strawberry genes in freezing stress tolerance, including the validation of gene modulation across independent experimental repetitions and the functional validation of candidate genes by knockdown/overexpression systems. Moreover, the analysis of hormonal and metabolic profiles (e.g., targeted metabolomics) and physiological and biochemical assays (e.g., enzyme activity and osmolyte accumulation) will further validate the mechanisms of freezing stress mitigation in bacterium-inoculated plantlets. Moreover, validation under field conditions is required to assess the efficacy of treatments using cold-adapted bacteria as a complementary tool to existing chemical and physical strategies to improve freezing stress tolerance in Rosaceae crops.

## Data Availability

Raw RNA reads were deposited at the Sequence Read Archive of the NCBI (https://www.ncbi.nlm.nih.gov/sra) under the BioProject number PRJNA1150759.
