# Supplementary material for: Cold-tolerant bacteria from alpine Rosaceae plants modulate transcriptional responses of apple and strawberry plantlets to freezing stress
Source: Front Plant Sci. 2026 Jun 26;17:1843975. doi: 10.3389/fpls.2026.1843975 (PMC13349782; doi:10.3389/fpls.2026.1843975)
Supplement: Supplementary file 1 [file DataSheet1.pdf]

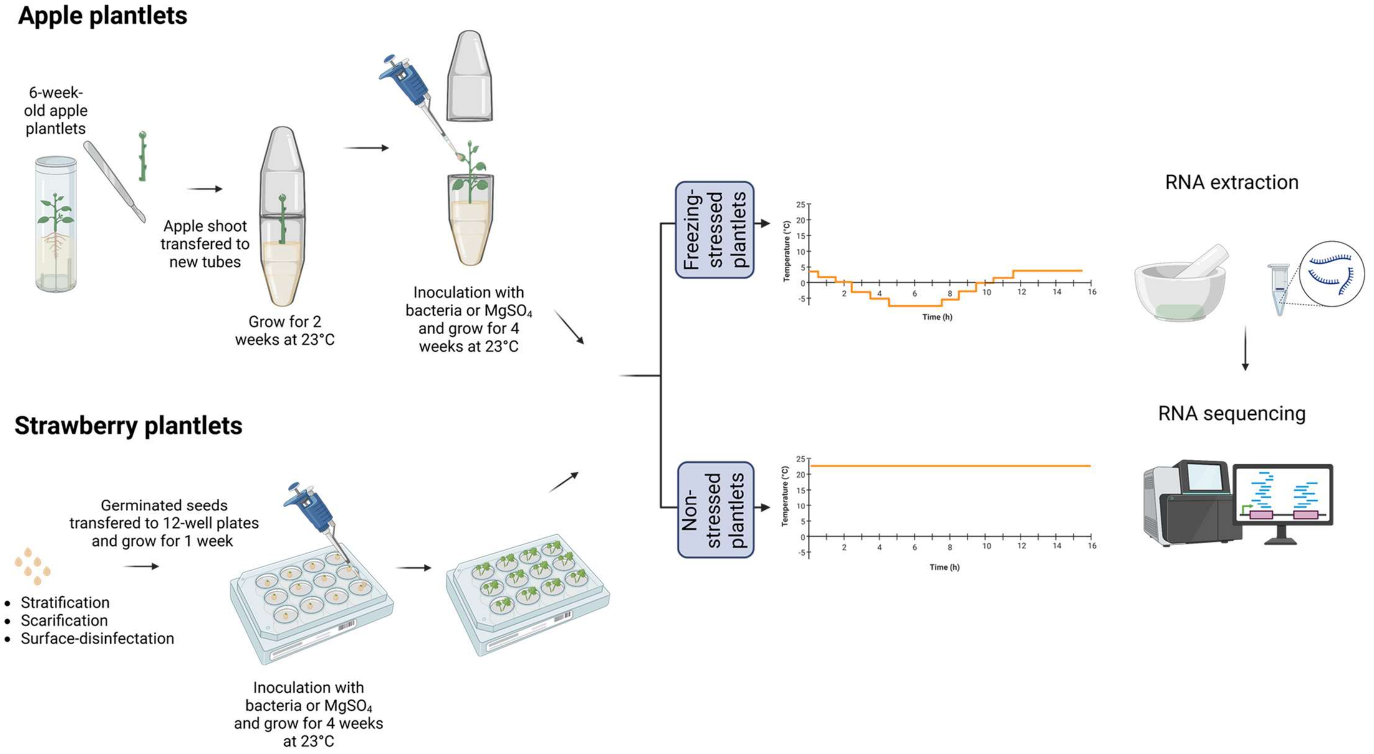

Supplementary Figure S1. Experimental design to study freezing stress response of apple and strawberry plantlets inoculated or not with the cold-tolerant bacterial endophytes *in vitro*.

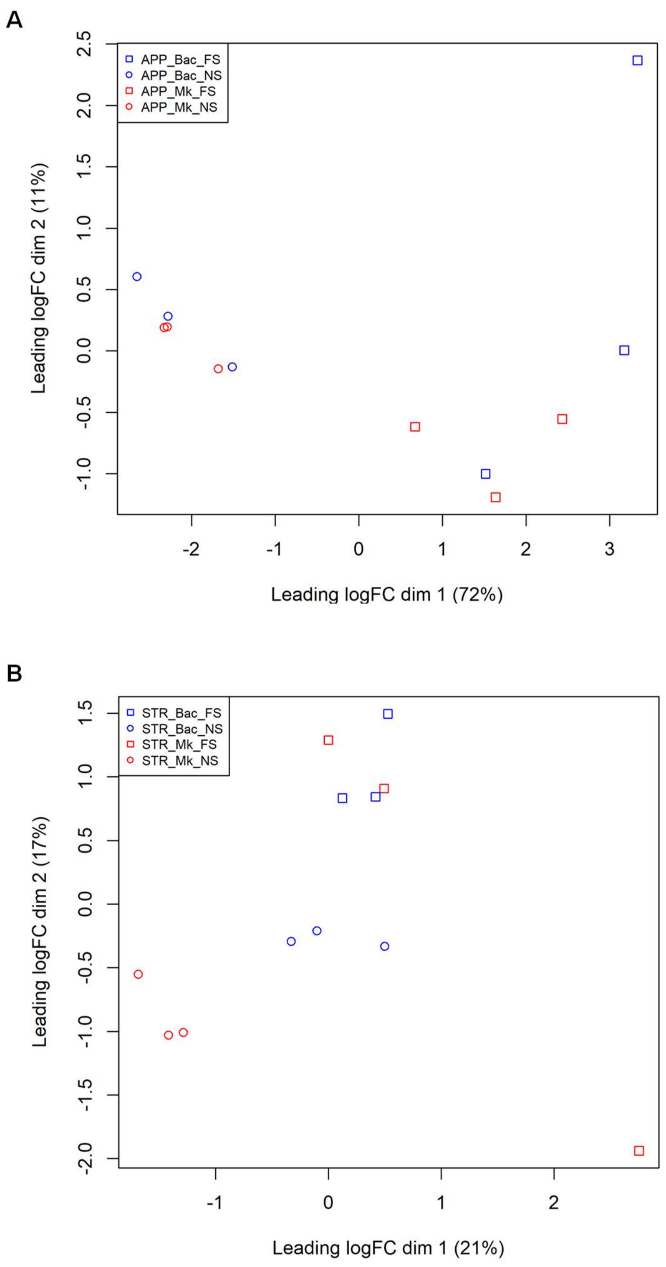

Supplementary Figure S2. Multi-dimensional scaling analysis (MDS) of the normalized read counts of the RNA-seq libraries generated from shoot samples of apple (A) and strawberry (B) plantlets treated with 10 mM $MgSO_4$ (mock-inoculated; Mk) or inoculated (bacterium-inoculated; Bac) with *Pseudomonas* GRAN103 and *Duganella* ALCN104, respectively, and exposed (freezing-stressed, FS) or not (non-stressed, NS) to freezing stress.

Supplementary Table S1. List of cold-tolerant bacterial endophytes tested.

Fifteen cold-tolerant bacterial endophytes previously isolated from different tissues of alpine Rosaceae plants were selected according to their ability to mitigate freezing stress (best performing isolates in terms of reduction in electrolyte leakage) on strawberry seedlings (Marian et al., 2025 10.1128/mbio.01418-24).

Supplementary Table S2. RNA-seq data elaboration statistics for apple (A) and strawberry (B) samples.

RNA-Seq results are reported for apple (APP) and strawberry (STR) plantlets treated with 10 mM $MgSO_4$ (mock-inoculated; Mk) or inoculated (bacterium-inoculated; Bac) with *Pseudomonas* GRAN103 and *Duganella* ALCN104, respectively, and exposed (freezing-stressed; FS) or not (non-stressed; NS) to freezing stress. Shoots were collected in triplicate (numbered from 1 to 3) after exposure to freezing stress. The number of paired-end raw reads (column F), the number and the percentage of filtered paired-end reads after quality filtering (filtered read pairs; columns G and H), and the number and percentage of paired-end reads mapping to the apple genome (*Malus x domestica* GDDH13v1.1) and the strawberry genome (*Fragaria* x *ananassa* Camarosa Genome Assembly v1.0) are reported for each sample, indicating read pairs aligned in the expected orientation and within the expected distance (insert size) from each other (Align Concordantly).

Supplementary Table S3. Expression levels of apple transcripts.

For each apple transcript (*Malus x domestica* GDDH13 v1.1) expression levels are reported as raw counts for each sample of apple shoots treated with 10 mM $MgSO_4$ (mock-inoculated; Mk) or inoculated (bacterium-inoculated; Bac) with *Pseudomonas* GRAN103 and exposed (freezing-stressed; FS) or not (non-stressed; NS) to freezing stress in triplicate (numbered from 1 to 3).

Supplementary Table S4. Expression levels of strawberry transcripts.

For each strawberry transcript (*Fragaria x ananassa* Camarosa v1.0) expression levels are reported as raw counts for each sample of strawberry shoots treated with 10 mM $MgSO_4$ (mock-inoculated; Mk) or inoculated (bacterium-inoculated; Bac) with *Duganella* ALCN104 and exposed (freezing-stressed; FS) or not (non-stressed; NS) to freezing stress in triplicate (numbered from 1 to 3).

Supplementary Table S5. Differential expression analysis results.

Apple (APP) and strawberry (STR) plantlets were treated with 10 mM $MgSO_4$ (mock-inoculated; Mk) or inoculated (bacterium-inoculated; Bac) with *Pseudomonas* GRAN103 and *Duganella* ALCN104, respectively, and exposed (freezing-stressed; FS) or not (non-stressed; NS) to freezing stress. Differential expression analysis was carried out on active transcripts (RPM >1 in at least two libraries) with the likelihood ratio test imposing a Log_2-transformed fold change (LFC) lower than -2 or higher than 2 and a false discovery rate (FDR) lower than 0.05 for each pairwise comparison. For each plant species, the number of downregulated transcripts, upregulated transcripts, and total modulated transcripts is reported in the pairwise comparisons of mock-inoculated freezing-stressed plantlets and mock-inoculated non-stressed plantlets (MkFS *vs.* MkNS) and bacterium-inoculated freezing-stressed plantlets and bacterium-inoculated non-stressed plantlets (BacFS *vs.* BacNS).

Supplementary Table S6. Expression levels and functional annotations of differentially expressed transcripts (DETs) in apple plantlets.

Apple plantlets were treated with 10 mM $MgSO_4$ (mock-inoculated; Mk) or inoculated with *Pseudomonas* GRAN103 (bacterium-inoculated; Bac) and exposed (freezing-stressed; FS) or not (non-stressed; NS) to freezing stress. Differentially expressed transcripts (DETs) were selected imposing a Log2-transformed fold change (LFC) lower than -2 or higher than 2 and a false discovery rate (FDR) lower than 0.05 with the likelihood ratio test in four pairwise comparisons: i) mock-inoculated freezing-stressed plantlets and mock-inoculated non-stressed plantlets (MkFS *vs.* MkNS), ii) bacterium-inoculated freezing-stressed plantlets and bacterium-inoculated non-stressed plantlets (BacFS *vs.* BacNS), iii) bacterium-inoculated non-stressed plantlets and mock-inoculated non-stressed plantlets (BacNS *vs.* MkNS), and iv) bacterium-inoculated freezing-stressed plantlets and mock-inoculated freezing-stressed plantlets (BacFS *vs.* MkFS).

Groups of DETs were defined according to the Venn diagrams, such as transcripts upregulated (group 1) or downregulated (group 4) by freezing stress only in mock-inoculated plantlets, transcripts upregulated (group 2) or downregulated (group 5) by freezing stress only in bacterium-inoculated plantlets, and transcripts upregulated (group 3) or downregulated (group 6) by freezing stress in both inoculation conditions. For each transcript, the best hit of the NCBI-nr database and Swissprot database (as indicated on the GDR website) is reported. Transcripts were classified into 14 functional categories according to the annotation based on the protein descriptions.

Supplementary Table S7. Expression levels and functional annotations of differentially expressed transcripts (DETs) in strawberry plantlets.

Strawberry plantlets were treated with 10 mM $MgSO_4$ (mock-inoculated; Mk) or inoculated with *Duganella* ALCN104 (bacterium-inoculated; Bac) and exposed (freezing-stressed; FS) or not (non-stressed; NS) to freezing stress. Differentially expressed transcripts (DETs) were selected imposing a Log2-transformed fold change (LFC) lower than -2 or higher than 2 and a false discovery rate (FDR) lower than 0.05 with the likelihood ratio test in four pairwise comparisons: i) bacterium-inoculated non-stressed plantlets and mock-inoculated non-stressed plantlets (BacNS *vs.* MkNS), ii) bacterium-inoculated freezing-stressed plantlets and mock-inoculated freezing-stressed plantlets (BacFS *vs.* MkFS), iii) mock-inoculated freezing-stressed plantlets and mock-inoculated non-stressed plantlets (MkFS *vs.* MkNS), and iv) bacterium-inoculated freezing-stressed plantlets and bacterium-inoculated non-stressed plantlets (BacFS *vs.* BacNS).

Groups of DETs were defined according to the Venn diagrams, such as transcripts upregulated (group 1) or downregulated (group 4) by freezing stress only in mock-inoculated plantlets, transcripts upregulated (group 2) or downregulated (group 5) by freezing stress only in bacterium-inoculated plantlets, and transcripts upregulated (group 3) or downregulated (group 6) by freezing stress in both inoculation conditions. For each transcript, the best hit of the NCBI-nr database and Swissprot database (as indicated on the GDR website) is reported. Transcripts were classified into 14 functional categories according to the annotation based on the protein descriptions.
